# Developmental dysplasia of the hip in infants referred for a combined pediatric orthopedic and radiologic examination. A prospective cohort study

**DOI:** 10.1016/j.jor.2022.05.014

**Published:** 2022-05-24

**Authors:** Simon Norlén, Christian Faergemann

**Affiliations:** a Section for Pediatric Orthopaedics, Department of Orthopaedics and Traumatology, Odense University Hospital, J.B. Winslow Vej 4, DK-5000, Odense C, Denmark; b Orthopaedic Research Unit, Department of Clinical Research, Faculty of Health Sciences, University of Southern Denmark, J. B. Winslow Vej 4, DK-5000, Odense C, Denmark

**Keywords:** Infant, Developmental dysplasia of the hip, DDH, Screening

## Abstract

Introduction: The study aimed to determine the proportions of infants with developmental dysplasia of the hip (DDH) and hip dislocation in infants referred for combined pediatric orthopedic and radiologic assessment, and to describe the association between DDH and different reasons of referral.

Methods: A prospective study of all infants aged 0-6 months referred for a combined examination of the hips 2013-2019. The proportion of DDH and unstable hip(s) stratified by different reasons of referral were calculated. Acetabular index > 30° in radiography or Graf Type 2b or worse in ultrasonography was considered diagnostic of DDH.

Results: Of the 1,989 infants included, 17% had stable dysplastic hip(s), and 4.7% had unstable dysplastic hip(s). The proportions of infants with DDH among infants with a single reason of referral were 36% for breech position, 25% for familial disposition, 14% for hip click, 8% for asymmetry, and 3% for twins. The proportions of infants with unstable hip(s) were 14% for familial disposition, 12% for breech position, 3% for hip click, 3% for twins, and 1% for asymmetry.

Conclusion: The study demonstrates that a considerable proportion of infants referred for the combined examination have radiological signs of DDH and that DDH were regularly diagnosed in infants referred due to hip click or asymmetry.

## 1 Introduction

Developmental dysplasia of the hip (DDH) encompasses a wide spectrum of anatomically abnormal hip developments, ranging from mild acetabular dysplasia to frank dislocation.^1^ The incidence of DDH has been reported to range from 6.6 to 8 per 1000 newborns in Western studies.^2^^,^^3^

Recommendations for treatment of DDH are based on both the clinical hip examination and imaging.^1^ Early referral allows treatment of unstable hips with bracing or casting.^1^ Early treatment prevents long-term hip dysplasia, hip abnormalities, and arthritis with complaints like impaired walking and chronic pain in hips, knee and lower back, requiring reconstructive surgery or hip replacement.^1^^,^^4^ Screening programs for DDH usually involves clinical examination in the neonatal period and during well-child consultation, ultrasound examination (universal or selective) or a combination or both.^1^^,^^4^

To detect hip abnormalities early, all newborns in Denmark are examined postnatally by a pediatrician and by a primary care physician at respectively five weeks and five months of age. Infants with persistent breech presentation, familial history of DDH and twins and infants with suspect findings such a hip click or hip asymmetry are referred to a combined pediatric orthopedic examination and ultrasonography (age <6 months) or radiography (age ≥6 months). Based on available literature and consensus, the XXXX pediatric orthopedic physicians have defined the screening policy. The Danish public health insurance covers the combined examination and treatment of DDH. This combined examination is resource demanding for the public health care system, and studies have found variating effects of such screenings.^4^, ^5^, ^6^ Furthermore, previous studies have described variation in the proportion of infants with DDH and unstable hip(s), among infants referred for the combined examination.^7^, ^8^, ^9^, ^10^ Additionally, only few studies have examined which causes most commonly lead to referral, and which causes frequently are associated with DDH.^7^^,^^10^

The aim of this study is to determine the proportion of infants with DDH and unstable hip(s) in all infants referred for combined pediatric orthopedic and radiologic assessment, and to describe the association between DDH and the different reasons of referral.

## 2 Methods

This study is a prospective cohort study. The cohort consist of infants referred to a combined pediatric orthopedic and radiologic examination for DDH in the Region of Southern Denmark (RSD) 2013–2019. RSD is a geographically well-defined region with a population of 1.2 million (21% of the total population in Denmark).^11^ In the study period 2013–19 there were 80,772 livebirths in RSD.^11^ In 2013 and 2019 the numbers were 11,303 and 11,582, respectively.^11^ The pediatric orthopedic services at Odense University Hospital (OUH) and Kolding Hospital (KH) are the only pediatric orthopedic services in the region. The same small group of pediatric orthopedic physicians cover the services at both hospitals.

All infants aged 0–6 months inhabited in the RSD and referred to OUH or KH for a combined pediatric orthopedic and radiographic examination for DDH from January 1st^,^ 2013 to December 31st^,^ 2019 were included. The infants were included prospectively and consecutively, as they met for examination. All infants were examined within 2–4 weeks after referral. Parents failing to show up for examination were contacted by telephone and/or by letter addressing the importance of the examination. Infants, whose parents rejected examination, were excluded.

From the medical journals and by interviewing the parents information about the birth, reasons for referral, referring unit, results of clinical examination, diseases/deformities, and treatment were obtained. Additionally, we obtained the results of radiological measurements (ultrasonography or radiographs). Reasons of referral were grouped into the following categories: hip click, asymmetry, familial disposition, breech position, twin birth, and other/unspecified. Asymmetry covered both asymmetrical skinfolds on thighs or glutes, leg length discrepancy, and unilateral limitation of hip abduction. Familial disposition was limited to first-degree relatives (parents and siblings). Breech position was defined as delivery in breech position.

The referred infants were examined by an experienced pediatric orthopedic specialist for positive clinical findings including Ortolani and Barlow maneuvers, Galeazzi's sign, restricted abduction of the hips, and asymmetry. Subsequently, ultrasonographical examinations were performed by an experienced radiologist. In few cases of infants aged close to 6 months, a plain radiograph was performed instead of sonography. On ultrasonography, DDH were defined as Graf's classification type 2b or worse (α-angle ≤ 59°) and/or coverage of ≤50% of femoral head.^12^ Diagnostic criteria for DDH on radiography were defined as an acetabular index (AI) ≥ 30°.^13^ Visibly dislocated hips on radiograph was also considered diagnostic of DDH.

**Fig. 1:**
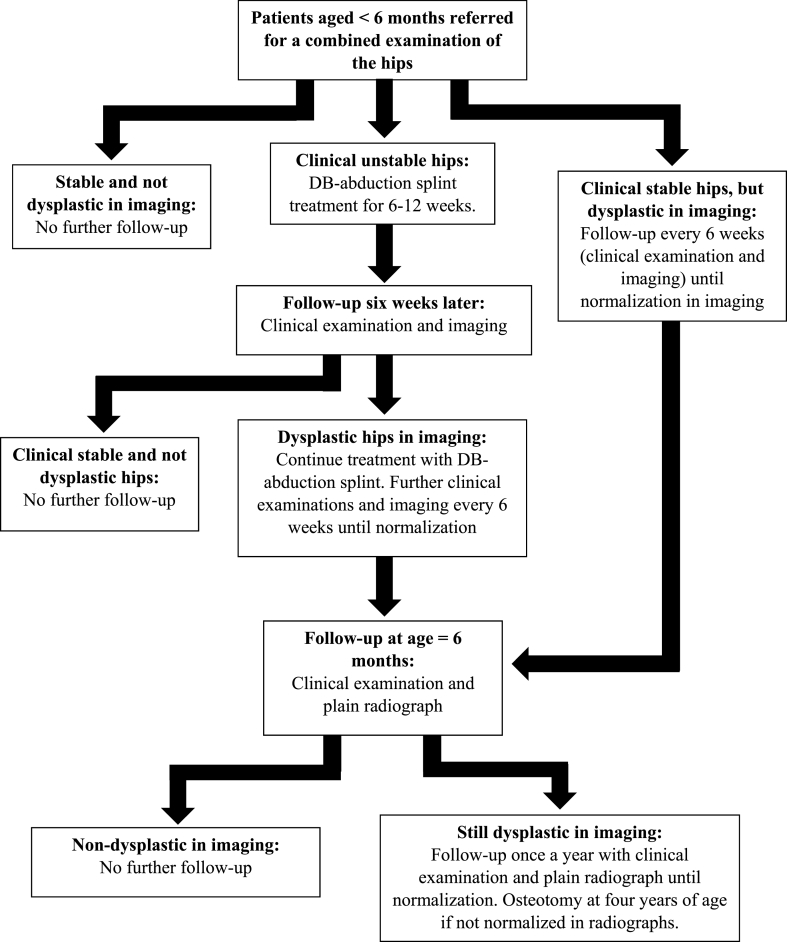
Algorithm for examination and treatment of DDH in Region of Southern Denmark.

Fig. 1 shows the algorithm for examination and treatment of DDH in RSD. In case of unstable/dislocated hip(s) (Ortolani or Barlow positive) the infant is treated with a Dennis Brown (DB) abduction splint for at least 6 weeks (until stability). Infants with stable but dysplastic hip(s) are followed up with ultrasonography and clinical examination every 6 week until the age of 6 months or normalization. From the age of 6 months the infants undergo clinical examination and radiographs once a year until radiological normalization. In case of dysplasia in the age of four years the children are offered an osteotomy.

The collected data were compiled and analyzed using EpiData Analysis Classic V2.2.3.187. The proportions of DDH and unstable hip(s) were calculated including 95% confidence intervals (CI). For infants with only one reason of referral, the proportions of infants with DDH and unstable hip(s) were calculated stratified by reason of referral. For infants with two or three reasons of referral, the proportions were calculated for every combination of referral reasons. A flowchart of the results and treatment of the first four combined examinations in each infant was constructed.

## 3 Results

During the 7-year study period, 1,989 infants (60% girls) aged ≤6 months were referred for a combined examination and thus included in the study. The mean age at time of the first examination was 61 days (range 3–180). Most infants (52%) were referred from their general practitioner, and 62% were referred to OUH (Table 1).

**Table 1:** The referring units, hospital of examination, gender, patient characteristics, information on births, and imaging methods.

*Referring unit*		N	Proportion
	General practitioner Obstetric unit Pediatric unit Other/unknown	1,034 932 10 13	52% 47% <1% <1%
*Hospital*	OUH KH	1,225 764	62% 38%
*Gender*	Girls Boys	1,194 795	60% 40%
*Birth*	Vaginal Cesarean section	1,568 421	79% 21%
*Gestational age*	<37	144	7%
*(weeks)*	37–42 >42	1,830 15	92% <1%
*Birthweight (gram)*	<3000 3000–4000 >4000	343 1,347 299	17% 68% 15%
*Imaging method*	Ultrasonography Radiography Both	1,924 63 2	97% 3% <1%
*Total*		N = 1,989	100%

Overall, 334 (17%) infants were diagnosed with DDH based on imaging findings at the first examination corresponding to an incidence rate of 4.1 (C.I.: 3.7–4.6) per 1000 newborns/year. Overall, 221 (66%) of the infants had unilateral DDH, while 113 (34%) had bilateral DDH. In 325 (97%) infants DDH were diagnosed by ultrasonography, while in 9 (3%) DDH were diagnosed by radiograph.

**Fig. 2:**
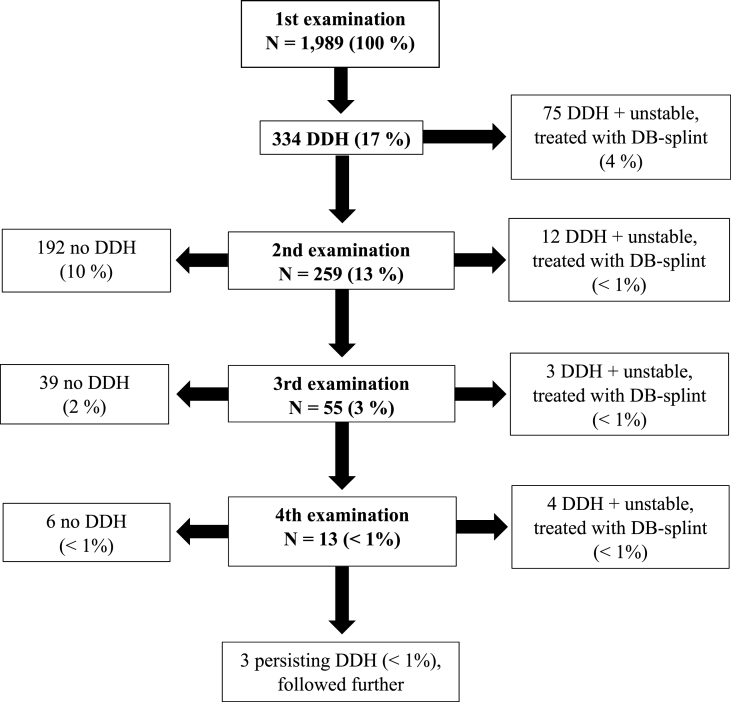
Flowchart of the results and treatments of the first four combined examinations of infant hips.

Overall, 94 (4.7%) infants had unstable hip(s) and required treatment corresponding to an incidence rate of 1.2 (C.I.: 0.9–1.4) per 1000 newborns/year. Fig. 2 shows the overall results and follow-up of the first four examinations. All percentages in the flowchart are of the total number of infants.

Table 2 summarizes the proportions of DDH and unstable hips stratified by reasons of referral. The number of infants with DDH include infants with unstable hip(s). Overall, 1,752 (88%) infants had one reason of referral, while 226 (11%) had two, and 11 (<1%) had three reasons of referral. The most common reasons of referral in any combination were hip click (63%), asymmetry (20%) and breach presentation (13%).

**Table 2:** Proportions of DDH and unstable hips in infants stratified by different reasons of referral. Only infants with one or two reasons of referral are shown.

	N	DDH^a^		Instability	
		n	Proportion (C.I)	n	Proportion (C.I)
**All infants**	1,989	334	0.17 (0.15–0.19)	94	0.05 (0.04–0.06)
**1 reason of referral**					
*Hip click*	1,091	156	0.14 (0.12–0.16)	32	0.03 (0.02–0.04)
*Asymmetry*	338	26	0.08 (0.05–0.11)	4	0.01 (<0.01–0.03)
*Breech position*	159	58	0.36 (0.30–0.44)	19	0.12 (0.07–0.18)
*Familial disposition*	104	26	0.25 (0.17–0.34)	15	0.14 (0.08–0.23)
*Other/unspecified*	31	5	0.06 (0.01–0.21)	1	0.03 (<0.01–0.17)
*Twin*	29	1	0.03 (0.01–0.18)	1	0.03 (<0.01–0.18)
**2 reasons of referral**					
*Breech position + Hip click*	56	17	0.31 (0.19–0.44)	6	0.11 (0.04–0.22)
*Familial disposition + Hip click*	49	12	0.25 (0.13–0.39)	4	0.08 (0.02–0.20)
*Hip click + Asymmetry*	30	4	0.13 (0.04–0.31)	0	0
*Hip click + Twin*	25	3	0.12 (0.03–0.31)	1	0.04 (0.01–0.20)
*Breech position + Familial disposition*	19	8	0.42 (0.21–0.67)	5	0.26 (0.09–0.51)
*Breech position + Twin*	15	6	0.40 (0.16–0.68)	2	0.13 (0.02–0.40)
*Breech position + Asymmetry*	12	5	0.42 (0.15–0.72)	2	0.17 (0.02–0.48)
*Familial disposition + Asymmetry*	9	4	0.44 (0.14–0.79)	2	0.22 (0.03–0.60)
*Familial disposition + Twin*	7	1	0.14 (0.04–0.58)	0	0
*Asymmetry + Twin*	3	0	0	0	0

a The number of infants with DDH includes those with unstable hip(s).

For single reasons of referral, the highest proportions of infants with DDH were among breech position and familial disposition, respectively 0.36 (95% CI [30–0.44]) and 0.25 (95% CI [0.17–0.34]). For infants solely referred due to hip click or asymmetry, the proportions of infants with DDH were respectively 0.14 (95% CI [0.12–0.16]) and 0.08 (95% CI [0.05–0.11]). The highest proportions of infants with unstable hip(s) were also among those referred due to breech position 0.12 (95% CI [0.07–0.18]) and familial disposition 0.14 (95% CI [0.08–0.23]). For hip click and asymmetry the proportions were respectively 0.03 (95% CI [0.02–0.04[) and 0.01 (95% CI [<0.01–0.03[). Among infants with two reasons of referral, the highest proportion of DDH were among those referred due to familial disposition and asymmetry 0.44 (95% CI [0.14–0.79]) and highest proportion of unstable hips were among those referred du e to breech position and familial disposition 0.26 (95% CI [0.09–0.51[).

Infants with three reasons of referral are not included in Table 2 due to very low numbers of infants in each combinations. Two of 11 (0.18, 95% CI [0.02–0.52[) infants were diagnosed with DDH though. One was referred with hip click, familial disposition, and breach position, and one was referred with hip click, familial disposition and asymmetry. None of the infants had unstable hip(s).

## 4 Discussion

Overall, this prospective study found that 17% of the infants fulfilled the diagnostic criteria for DDH by either ultrasonography or radiography. Similar proportions were found in studies from Ireland and Hong Kong.^4^, ^5^, ^6^, ^7^, ^8^, ^9^, ^10^ The overall proportion of DDH in our study corresponds to an incidence rate of 4.1 per 1000 newborns/year, which corresponds to population-based incidence rates of DDH in other studies.^2^^,^^3^ However, the estimated incidence rate in our study only includes infants referred for the combined examination, and is not based on examination of all infants in RSD. Therefore, the population-based incidence rates of DDH among all newborns in RSD are expected to be higher.

The overall proportion of infants with unstable hip(s) was 4.7%, corresponding an incidence rate of 1.2 per 1000 newborns/year. A Norwegian study of newborns found, that 0.8% had dislocated or unstable hips at the initial clinical examination.^14^ In a UK study of infants aged < nine weeks referred with suspected instability or recognized risk-factors, the rate of instability of the hip was 2.1 per 1000 live births.^8^ It is noteworthy, that the Norwegian study examining an unselected population found a higher incidence rate than in our study and the English study.

Hip click was the most common single reason of referral. Of the 1,091 infants referred only with ‘hip click’, 14% were diagnosed with DDH, and 3% required treatment due to unstable hip(s). The significance of hip click remains controversial in the literature. A study by Bond et al. examined 50 neonates with isolated hip clicks, and found no hip abnormalities at reevaluation at 3 months of age, concluding that soft tissue hip clicks were not abnormal.^15^ A study by Kamath et al. evaluated 176 infants aged >6 weeks with isolated hip click, and found no pathologic hips at 6-months reevaluation, concluding that inclusion of clicky hip as a risk factor in the targeted ultrasound screening will not reduce the incidence of missed cases.^16^ On the contrary, Groarke et al. found a positive predictive value (PPV) for DDH of 14.3% in children with hip click as reason of referral.^7^ Furthermore, Cunningham et al. demonstrated that hip instability was 39 times more frequent in infants with minor signs, like hip click, on examination within 48 h of birth, than infants considered normal, concluding that a clicky hip should never be ignored.^17^ Both last-mentioned studies support our findings, demonstrating that hip click referrals can represent underlying pathology, and should lead to further assessment.

Asymmetry was the second most common single reason of referral, covering both asymmetrical skinfolds on thighs or glutes, leg length discrepancy, and unilateral reduced hip abduction. Not analyzed separately, only 8% of the infants with asymmetry had DDH. In the study by Groarke et al., PPVs of DDH were 12.4% for asymmetrical skin folds, 23.5% for limb shortening, and 29.4% for reduced abduction.^7^ Other studies have also demonstrated the significance of reduced hip abduction in DDH. Choudry et al. found that unilateral limited abduction of the hip had a PPV of 40% for DDH, while bilateral limited abduction had a PPV of only 0.3%, suggesting that the presence of bilateral limited abduction in the infant may be a normal variant, while unilateral limited abduction is an important clinical sign, which should be actively sought.^18^ This finding is supported by Sewell et al.^19^ A systematic review by Shipman et al. found it difficult to conclude, that findings of asymmetrical skin folds and leg-length discrepancy had any usefulness, based on the scarce literature.^6^ Collectively, these studies suggest, that the different clinical signs of asymmetry are of different diagnostic value. Unfortunately, we have not been able to analyze the different factors of asymmetry separately.

The two single reasons of referral with the highest proportions of infants with DDH in our study were breech position (36%) and familial disposition (25%). Among these infants 12% and 14% required treatment due to clinically unstable hip(s). A study from Switzerland of screened newborns found statistically significant odds ratios of 4.98 for breech presentation and 5.05 for family anamnesis as risk factors for DDH at age 2–5 days.^20^ Other studies also support breech position and familial disposition as important risk factors for DDH.^3^^,^^8^

The study demonstrates that a considerable number of infants with stable, dysplastic hip(s) normalized spontaneously. This support that active surveillance can reduce the need for treatment without increasing the risk of persisting DDH. This is also supported by other studies.^6^^,^^21^ A study of Rosendahl et al. showed that active-sonographic-surveillance halved the number of children requiring treatment, without increasing the duration of treatment.^21^ In our study 19 infants with stable, dysplastic hip(s) at the first examination, were found to be unstable requiring treatment at later examinations. This supports the importance of surveillance of infants with dysplastic hip(s).

In our study no infants were reffered due to clubfeet or torticollis. However, we found 7 infants with clubfeet (1 with DDH) and 18 infants with torticollis (4 with DDH) in the study group. Previous studies have found torticollis as a significant risk factor of DDH, while the significance of clubfeet remains controversial in literature.^3^^,^^8^^,^^22^^,^^23^

The diagnostic criteria for DDH were based on commonly used methodologies in literature.^7^^,^^20^^,^^24^^,^^25^ In radiography, some studies use values > 2 standard deviations (SDs) higher than the reference values for the age, as published by Tönnis et al.^13^^,^^19^^,^^24^ Lower thresholds for diagnosis increase the risk of overtreatment, and hereby expose otherwise healthy children to iatrogenic complications, while higher threshold values increase the risk of late presenting DDH and delayed treatment. Despite the screening program in RSD, some children may have a late diagnose of DDH or not diagnosed until the present of hip pain later in life. So far, we have no reliable data regarding late presenting DDH in older children or in adults. In a single study from RSD, 16 children were diagnosed with hip dislocation due to DDH later than six months of age over a three year period.^26^ All children had been routinely screened but not referred for the combined examination.

According to our treatment protocol, we use the static DB-splint for the treatment of unstable dysplastic hips, which is the standard treatment in Denmark, and common choice of treatment in other parts of Scandinavia. Although, the dynamic bracing with Pavlik harness is thought of as the standard and uniform treatment, a study have shown that about 20% of European and North American pediatric orthopedic specialists prefer static bracing for treatment of unstable dysplastic hips.^27^ In a systematic review has described high rates of efficiency and low rates of complications in both static and dynamic bracing of dysplastic hips.^28^

The strength of our study is the prospective and consecutive design in a well-defined geographic area. No other hospitals or clinics in the region have pediatric orthopedic services. In the study period, only four infants’ parents rejected the combined orthopedic pediatric and radiologic examination of their child. Another strength is that only four different highly experienced radiologists and eight different pediatric orthopedic specialists examined all the infants in the study. A previous study has shown the importance of the experience of the examiner.^5^ In Denmark, all medical examinations and treatments are covered by the public health insurance. Therefore, the combined examinations for DDH is free of charge for all. No parents turned down the combined examination due to financial issues, and all referred infants/parents showed up for the examinations.

A screening program for DDH in newborns and a combined orthopedic and radiologic examination of infants with risks factors or symptoms of DDH are highly recommendable. The present study shows considerable proportion of infants referred for this combined examination have radiological signs of DDH and some need early treatment due to instability of the hip(s). Future studies should assess the cases of missed DDH to determine the effect of the screening protocol.

Our study also indicates that newborns with not only familial disposition or breech position should be referred to a combined orthopedic and radiologic examination, but also newborns with hip clicks or asymmetry need this combined examination. Since we have limited data regarding late presenting DDH, we cannot conclude if the criteria for referring infants to a pediatric orthopedic unit should be changed in order to increase the effectiveness of the protocol. Further studies should assess the number of late presenting cases, also in the subset of patients where the hips was stable and normal in imaging at the combined examination. Also, our study only includes infants referred for the combined examination, and not all newborns in the region. Future studies of all newborns could contribute to more exact estimates of the incidence and treatment rates of DDH. Additionally, future studies should examine the need for surgical procedures later in the infants’ lives or in adulthood among the infants, who received treatment (DB-splint), and infants with persisting DDH after the fourth examination. Further studies should include these infants for to find their longer-term outcomes.

## 5 Conclusion

Our results demonstrates that a considerable proportion of infants referred for the combined orthopedic and radiologic examination have radiological signs of DDH. Furthermore, the study

Demonstrates that a considerable number of infants with stable, dysplastic hip(s) normalized spontaneously before the age of year. The highest proportion of DDH with or without instability of the hip(s) were among infants referred due to familial disposition, breech position or a combination of both. However, DDH were also regularly diagnosed in infants referred due to hip click or asymmetry.

## Ethical committee approval

The study received approval from the RDS (Journal number 17/38417). Due to the large number of participants, informed consent was obtained from the Danish Patient Safety Authority (Journal number 3-3013-2434/1).

## Funding

None.

## CRediT authorship contribution statement

**Simon Norlén:** Formal analysis, Investigation, Data curation, Writing – original draft, Project administration.

**Christian Faergemann:** Conceptualization, Methodology, Software, Resources, Validation, Writing – review & editing.

## Declaration of competing interest

None.
